# Glycoprotein α-Subunit of Glucosidase II (GIIα) is a novel prognostic biomarker correlated with unfavorable outcome of urothelial carcinoma

**DOI:** 10.1186/s12885-022-09884-8

**Published:** 2022-07-25

**Authors:** Qiongqiong Lin, Lu Pei, Zhiguang Zhao, Xiaoping Zhuang, Haide Qin

**Affiliations:** 1 Department of Pathology, the Second Affiliated Hospital & Yuying Children’s Hospital of Wenzhou Medical University, Wenzhou, Zhejiang China; 2 Guangdong Provincial Key Laboratory of Malignant Tumor Epigenetics and Gene Regulation, Sun Yat-Sen Memorial Hospital, Sun Yat-Sen University, 510120 Guangzhou, China; 3 Department of Pathology, Wenzhou Hospital of Integrated Traditional Chinese and Western Medicine, Wenzhou, Zhejiang China; 4 Department of Urology, Sun Yat-Sen Memorial Hospital, Sun Yat-Sen University, Guangzhou, China

**Keywords:** Glycosylation, Glucosidase, Post-translational modifications (PTMs), GANAB, Urothelial Carcinoma (UC), Stress granules (SGs)

## Abstract

**Background:**

Urothelial carcinoma (UC) is among the most prevalent malignancies. The muscle-invasive bladder cancer (MIBC) shows an invasive feature and has poor prognosis, while the non-muscle invasive bladder cancer (NMIBC) shows a better prognosis as compared with the MIBC. However, a significant proportion (10%–30%) of NMIBC cases progress to MIBC. Identification of efficient biomarkers for the prediction of the course of UC remains challenging nowadays. Recently, there is an emerging study showed that post-translational modifications (PTMs) by glycosylation is an important process correlated with tumor angiogenesis, invasion and metastasis. Herein, we reported a data-driven discovery and experimental validation of GANAB, a key regulator of glycosylation, as a novel prognostic marker in UC.

**Methods:**

In the present study, we conducted immunohistochemistry (IHC) assay to evaluate the correlation between the expression levels of GANAB protein and the prognosis of UC in our cohort of 107 samples using whole slide image (WSI) analysis. In vitro experiments using RNAi were also conducted to investigate the biological functions of GANAB in UC cell lines.

**Results:**

We observed that positive GANAB protein expression was significantly correlated with poor prognosis of UC in our cohort, with p-value of 0.0017 in Log-rank test. Notably, tumor cells at the invasive front of the tumor margin showed stronger GANAB expression than the tumor cells inside the tumor body in UCs. We further validated that the elevated expression levels of GANAB were significantly correlated with high grade tumors (p-values of 1.72 × 10^–10^), advanced stages (6.47 × 10^–6^), and elevated in luminal molecular subtypes. Moreover, knocking-down GANAB using RNAi in UM-UC-3 and T24 cells inhibited cell proliferation and migration in vitro. Knockdown of GANAB resulted in cell cycle arrest at G1 phase. We demonstrated that GANAB mediated HIF1A and ATF6 transcriptional activation in the ER stress signaling, and regulated the gene expression of cell cycle-related transcriptional factors E2F7 and FOXM1.

**Conclusions:**

The elevated expression of GANAB is a novel indicator of poorer prognosis of UC. Our data suggests that GANAB is not only a new and promising prognostic biomarker for UC, but also may provide important cues for the development of PTM-based therapeutics for UC treatment.

**Supplementary Information:**

The online version contains supplementary material available at 10.1186/s12885-022-09884-8.

## Background

Urothelial carcinoma (UC) is among the most prevalent malignancies worldwide, which can present as non-muscle invasive bladder cancer (NMIBC) [1], and muscle-invasive bladder cancer (MIBC) [2]. The stage and grade are strongly correlated with the prognosis of UC. Great efforts had been made for the treatment of UC, however, tumor recurrence and progression continue to occur frequently in patients. UCs of the same morpologic type and stage might present different treatment outcome due to the significant intratumoral and intertumoral heterogeneity. The reported 5-year rates of recurrence for NMIBC range from 50 to 70%, and there is a significant proportion of NMIBC cases (10%–30%) progress to MIBC [3]. Currently, it remains difficult to predict the course of UC, which pose the challenges for clinical management. Therefore, identifying efficient prognostic molecular markers is crucial in the stratification of UC patients for individualized therapeutic strategies.

Recent studies showed that post-translational modification (PTM) pathways were correlated with tumor angiogenesis, invasion and metastasis [4, 5]. Protein glycosylation such as N-linked glycosylation or O-linked glycosylation, is one of the most important processes among the PTM pathways. It was reported that glycosylation related enzymes were critical for proper folding of proteins in endoplasmic reticulum (ER) stress process and stress granule (SG) formation [6, 7]. Previous studies revealed that the inhibition of ER stress could suppress tumorigenesis [8]. These literatures indicated that ER associated proteins and the glycosylation-related proteins might serve as therapeutic targets in cancers.

In our data-driven approach to analyze the prognostic genes of UC, we found that multiple PTM genes were significantly correlated with the prognosis of UC. Among these genes, we identified the elevated expression levels of GANAB were significantly correlated with the unfavorable outcome of UC. Previous studies have established that GANAB, also named α-subunit of glucosidase II (GIIα), is a key regulator of glycosylation. Khaodee et al*.* showed that it was essential for Glucosidase II (GluII) to regulate PTMs of N-linked glycoproteins in tumor progression [9]. GIIα catalyzes the trimming of the terminal glucose residues of N-glycan in glycoprotein processing coupled with quality control in the ER. Moreover, emerging literatures have reported that PTMs play a vital role in tumorigenesis and metastasis [10, 11]. However, no study has been reported to demonstrate the roles of GANAB expression in UCs, and the molecular mechanism has not been elucidated.

Herein, to investigate the roles of the N-linked glycoprotein GANAB in UC progression, we conducted immunohistochemistry (IHC) to show the expression of GANAB protein in our cohort of 107 UC samples using whole slide image (WSI) analysis. We observed that elevated GANAB protein expression levels were closely correlated with the high pathological grade, advanced stage and poor prognosis of UC. Moreover, functional studies further demonstrated that GANAB was involved in the regulation of UC cell proliferation, migration, along with the cell cycle. Our results suggest that GANAB is not only a novel prognostic biomarker for UC that contributes to the risk stratification, but also provide important cues for the development of PTM-based therapeutics for UC treatment.

## Methods

### Patients and tissue samples

The clinical information of study subjects have been described in one of our studies [12]. Briefly, a total of 107 UC samples with complete follow-up information were obtained for IHC at the Second Affiliated Hospital and Yuying Children’s Hospital of Wenzhou Medical University (WMU) between January 2014 and March 2020. Detail clinical pathological characteristics of patients with UC were summarized in Table 1. There was no significant difference between the age and gender distribution of the patients. Written informed consent was obtained from all patients, and the experimental protocols of all experiments were approved by the Ethical Committee of the Second Affiliated Hospital and Yuying Children’s Hospital of WMU (2021-K-101–01).

**Table 1:** Association test of the expression levels of GANAB and clinical variables

Variable	Sub-group	Negative-GANAB	Positive-GANAB	OR(95%CI)	*p*-value*
Sex	male	39(0.36)	47(0.44)		
	female	12(0.11)	9(0.08)		
				0.63(0.21–1.81)	0.343964
Age	≤ 60	13(0.12)	9(0.08)		
	> 60	38(0.36)	47(0.44)		
				1.78(0.62–5.26)	0.242931
Grade	low	38(0.36)	8(0.07)		
	high	13(0.12)	48(0.45)		
				16.91(6.03–53.13)	1.72 × 10^-10
Muscle Invasion (no:Ta + T1;yes: ≥ T2)	no	38(0.36)	25(0.23)		
	yes	5(0.05)	31(0.29)		
	na	8(0.07)	0(0)		
				9.2(3.01–34.47)	6.47 × 10^-6

^*^Fisher-exact test; Note, the missing group was excluded

### Immunohistochemistry assay

Serial sections (3 µm) of Formalin-fixed paraffin-embedded (FFPE) UC samples were stained on the Ventana BenchMark Ultra platform (Roche Diagnostics, Tucson). Immunostaining of sections are described in detail below. Deparaffinization, rehydration and incubating in Cell Conditioner 1 (prediluted; pH 8.0) for antigen retrieval for 30 min at 37˚C; Primary rabbit anti-GANAB monoclonal antibody was hand-applied and incubate for 32 min at 37˚C; Followed by applying UV HRP UNIV MULT (secondary antibody) for 8 min; Incubating in UV DAB and UV DAB H_2_O_2_ for 8 min; Counter-stained with hematoxylin II for 12 min and bluing reagent (Ventana) for 4 min, dehydrated and coverslipped. Rabbit monoclonal to GANAB (1:1500) was purchased from Abcam (ab176349). All slides were scanned as WSIs using the Nano Zoomer XR Digital Pathology microscope (Hamamatsu Photonics KK, Hamamatsu) at a magnification of 40 × and a resolution ratio of 512 × 512 pixels. Two pathologists independently evaluated the IHC staining.

### Immunohistochemistry evaluation

**Fig. 1:**
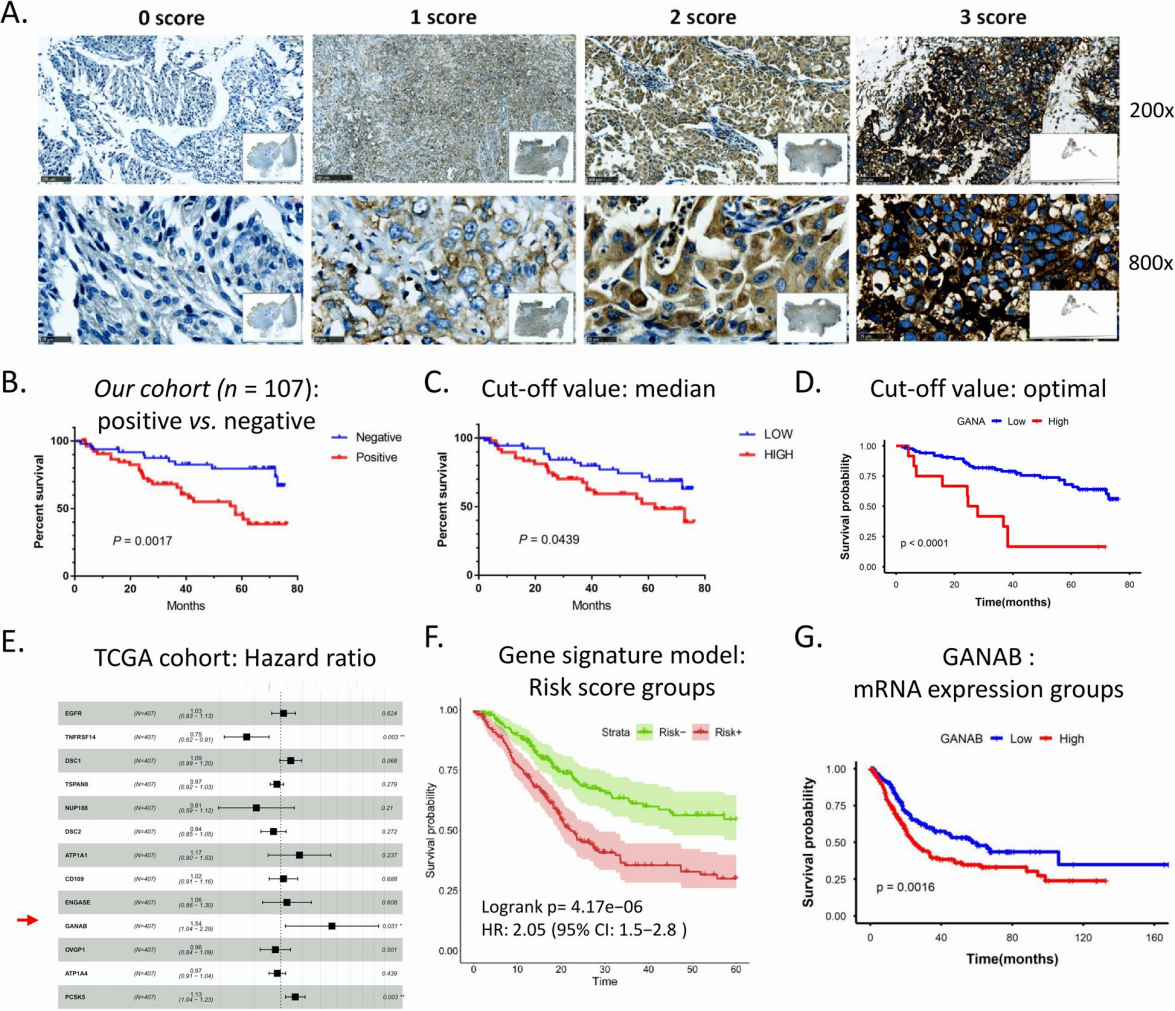
Elevated GANAB expression was significantly correlated with poor prognosis of UCs. **A**, Representative images to indicate the categorical IHC staining intensities. Score 0: Negative; Score 1: Weak staining; Score 2: Medium staining; Score 3: Strong staining. Scale bar: 100um; magnification at 200 × (top). Scale bar: 25μm; magnification at 800 × (bottom). **B**, The higher expression levels of GANAB was significantly correlated with poorer outcome of UCs in our cohort. Note, the patients with score 0 were grouped into the negative expression group; the patients with score 1–3 were grouped into the positive expression group. **C-D**, The high H-score group was significantly associated with the poorer survival of UCs in our cohort. The median cut-off value (**C**) and the optimal cut-off value (**D**) were used, respectively. **E**, Multivariate analysis of the Glycoprotein geneset using the Cox regression model based on filtered variables. **F**, Survival curves between high risk and low risk groups of the UC patients based on the risk scores in the Glycoprotein-based mRNA model. Log-rank test was used for the significant test. **G**, Survival curves between the high-expression and low-expression groups, stratified by the gene expression levels of GANAB in the TCGA-BLCA cohort. The optimal cut-off value of GANAB mRNA expression was used

A granular cytoplasmic staining pattern was considered as positive expression. IHC staining was  evaluated by the color intensity of positive tumor cells. The scoring approach for staining intensity were as follows: score 0 indicated no staining; score 1 indicated weak staining; score 2 indicated medium staining; and score 3 indicated strong staining (Fig. 1A). For the intensity group, score 0 represented negative, scores 1–3 were all defined as positive.

### Whole-slide image analysis

Qupath software [13] (version 0.2.1) was used to perform the automate quantification of H-scores [12]. Briefly, all original IHC slides were scanned and subjected to subsequent analyses. For each IHC slide, five representative Regions of Interest (ROIs) were selected by a pathologist using the Squared Tool (250 μm × 250 μm) in the Qupath software. A Groovy script was made for the whole-slide image analysis to automatically calculate the mean of H-scores for the ROIs on each slide.

### Cell culture

The UM-UC-3 and T24 cell lines used in this study were obtained from American Type Culture Collection (ATCC, Manassas, VA, USA). DMEM medium (GIBCO, Gaithersburg, MD, USA) was used for UM-UC-3 cells. T24 cells were cultured in RPMI-1640 medium (GIBCO, Gaithersburg, MD, USA). The culture medium contained 10% fetal bovine serum (FBS; Biological Industries, Beit Haemek, Israel) and 1% penicillin/streptomycin (GIBCO, Gaithersburg, MD, USA) and incubated in a humidified 5% CO_2_ atmosphere at 37℃. For induction of ER stress,Tunicamycin (2 µg/mL, MedChemExpress, USA) dissolved in dimethyl sulfoxide (DMSO) was added to the culture media in the presence or absence of siGANABs.

### RNA extraction and quantitative real-time PCR

RNAs from UC cells were isolated with RNAiso Trizol (Invitrogen, Shanghai, China). cDNAs were reversed transcribed using the PrimeScript™ RT reagent kit (Takara, Shiga, Japan). Quantitative real-time PCR (qRT-PCR) analysis was performed using TB Green Premix Ex TaqII (Takara, Shiga, Japan) in a Quantstudio Dx system (Applied Biosystems, Singapore) according to the manufacturer’s instructions. Results were normalized to the expression of glyceraldehyde 3-phosphate dehydrogenase (GAPDH), and the relative expression levels of mRNA were calculated by the 2 − ΔΔCt method. The primer (10 μM for each gene) sequences were as follows: GANAB, 5′- TGGGGATTACCCTTGCTGTG-3′(for), 5′- CCGTATGCTTCTCTGTCGCT-3′(rev); GAPDH, 5′-ACAACTTTGGTATCGTGGAAGG-3′(for), 5′-GCCATCACGCCACAGTTTC-3′(rev); HIF1A, 5′-GAACGTCGAAAAGAAAAGTCTCG-3′(for), 5′-CCTTATCAAGATGCGAACTCACA-3′(rev); FOXM1, 5′-ATACGTGGATTGAGGACCACT-3′(for), 5′-TCCAATGTCAAGTAGCGGTTG-3′(rev); ATF6, 5′-AGCAGCACCCAAGACTCAAAC(for), 5′-GCATAAGCGTTGGTACTGTCTGA-3′(rev); E2F7,5′-TAGCTCGCTATCCAAGTTATCCC-3′(for), 5′-CAATGTCATAGATGCGTCTCCTT-3′(rev).

### RNA interference

Small interfering RNA (siRNA) duplexes targeting GANAB were synthesized by GenePharma (Shanghai, China). Negative control siRNA sequences were purchased from the GenePharma. siRNAs were transfected by Lipofectamine RNAiMax (Life Technologies, Waltham, MA, USA), according to manufacturer’s recommendation. The sequences of the siRNAs were as follows: silencing control (siCtrl), UUCUCCGAACGUGUCACGUTT; siGANAB#1, GGGUUGAUAUAUCUUCCAATT; and siGANAB#2, CUGCGUCGAUUCUCAUUCUTT.

### Plasmid construction and transfection

The GANAB overexpression vector was constructed by using a pcDNA3.1 (+) vector (IGE Biotechnology, Guangzhou, China). Plasmid vectors were transfected into T24 and UM-UC-3 cells by X-tremeGENE HP DNA Transfection Reagent (Roche, Shanghai, China), following the manufacturer’s protocol.

### CCK-8 assay

T24 and UM-UC-3 cells transfected with siGANABs or pcDNA3.1(+)-GANAB were seeded in 96-well plates at a density of 1,000 cells/well and incubated at 37℃ for 6 days. 10 μL CCK-8 solution (Dojindo Laboratories, Kumamoto, Japan) was added to each well, followed by incubation for 2 h. Absorbance was measured at a wavelength of 450 nm on a Spark 10 M microplate reader (Tecan, Austria). Each assay was repeated in triplicate.

### Transwell assay

Cell migration and invasion abilities of UC cells was estimated by Transwell assay using Transwell chamber with pore size of 8.0 μm (Millipore, Darmstadt, Germany) according to the manufacturer’s instructions. 8 × 10^4^ UM-UC-3 cells or 6 × 10^4^ T24 cells were suspended in serum-free medium and plated on Transwell chambers with or without pre-coated Matrigel matrix (Corning, USA). The medium containing 10% FBS was added to the lower chamber as chemoattractant. After 20 h for UM-UC-3 cells or 8 h for T24 cells, the chambers were fixed in 4% paraformaldehyde (Servicebio, Wuhan, China) and then stained with 1% crystal violet solution (Sigma-Aldrich, Darmstadt, Germany) for 15 min and immersed in PBS for 10 min. Then, the migrated or invasive cells in the lower chamber membrane were observed and counted under an orthographic microscope (Nikon, Japan). The average cell numbers under five random fields of view (200 ×) were calculated. Three independent experiments were performed in the same conditions.

### Colony formation assay

Cells transfected with siRNAs or pcDNA3.1(+)-GANAB were seeded at a density of 1000 cells/well into six‑well plates. The cells were incubated for 14 days at 37˚C in a humidified incubator with 5% CO_2_. Subsequently, the colonies were fixed in 4% paraformaldehyde, stained with 0.1% crystal violet (Sigma-Aldrich) and imaged with vSpot Spectrum (AID, Germany). Data represent the average of three independent experiments.

### Cell cycle analysis

Cell cycle assay was performed by Cell Cycle Assay Kit (Elabscience, Wuhan, China) and measured following the protocols. T24 and UM-UC-3 cells treated with siGANABs or siCtrl were collected after 48h and then fixed with 70% ethanol at 4 ˚C overnight. The cells were washed twice before incubating for 30 min at 37℃ with 100 μL RNase A solution. At last, 400 μL propidium iodide (PI) solution was added to each flow's tube. Cell cycle was analyzed by CytoFLEX (Beckman,USA). The percentage of cells in each phage of the cell cycle was counted and compared.

### Bioinformatics analysis

Gene expression data and corresponding clinical information were downloaded from publicly available databases, including gene expression omnibus (GEO) datasets GSE3167, GSE38264 ( https://www.ncbi.nlm.nih.gov/geo/browse/?view=series), the Cancer Genome Atlas (TCGA, https://tcga-data.nci.nih.gov/tcga/), cBioPortal (URL: https://www.cbioportal.org), and the Human Protein Atlas (HPA) database (http://www.proteinatlas.org/). The SEEK bioinformatics tools was used to conduct co-expression analysis to identify the top-ranked genes with GANAB in bladder cancer datasets, including GSE32548, GSE31684 and GSE3167 (https://seek.princeton.edu/seek/). GSE129757 was used for analysis of ER stress related transcriptional factors induced by tunicamycin (Tm). Network analysis were conducted by using Cytoscape (Version: 3.9.1, https://cytoscape.org/).

### Statistical analysis

For visualization and Log-rank test, we used GraphPad Prism version 7 to generate the plots. We used *p*-value < 0.05 as significant level to interpretate the results. Correlation analysis was conducted using an in-house R script. Survival analysis was conducted using statistical R language based on packages including *survival, ggsurv,* and *survminer*. Robust likelihood-based survival modeling was concurred using R package *rbsurv*. Fisher-exact test was used for the association test between the expression levels of the gene with clinical variables. Comparisons between the two groups were performed using Student’s t-test and Mann-Whitney U test.

## Results

### GANAB is a novel prognostic factor in UCs

To explore whether GANAB expression could serve as a prognostic biomarker, we investigated the influence of GANAB on the prognosis of UC patients of our cohort (*n* = 107) by using survival analysis. The results indicated that the positive expression of GANAB were correlated with poorer prognosis of UCs compared with the negative expression levels of GANAB (*P*_log-rank_ = 0.0017) (Fig. 1B-D).

In the TCGA-BLCA dataset, univariate analysis of the glycoprotein genes ranked GANAB gene at the top of the unfavourable prognostic genes in UCs (Supplementary Table S1 and Supplementary Table S2). Multivariate analysis adjusted by other genes in the model also gave significant results for GANAB gene (Fig. 1E). Notably, we found that risk scores calculated using 13 glycoprotein-related genes incorporating GANAB were able to stratify the patients into two distinct risk groups (Fig. 1F); and the elevated GANAB was consistently correlated with the poor survival of UC (Fig. 1G). GANAB has not been reported to be associated with UCs previously in literature researches.

### Elevated expression levels of GANAB were correlated with high tumor grades

**Fig. 2:**
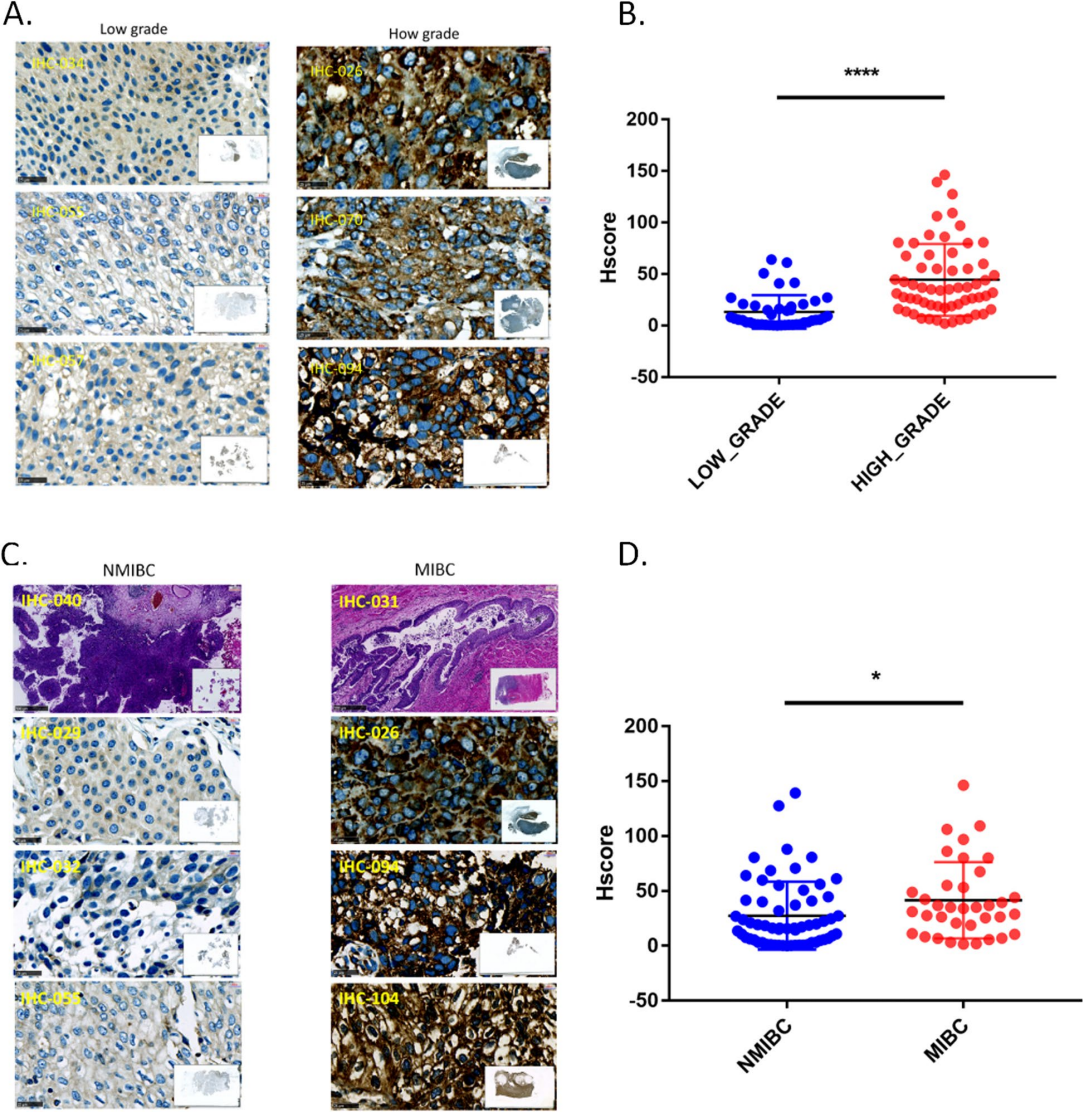
High histological grade and MIBC were correlated with elevated GANAB expression levels in UCs. **A-B**, The UCs of low-grade showed lower expression levels of GANAB, as compared with the UCs of high-grade. **C-D**, MIBC showed higher expression levels of GANAB as compared with NMIBC. IHC Scale bar: 25μm; IHC magnification at 800 × . HE Scale bar: 500μm; HE magnification at 50 × . IHC assay IDs were indicated in yellow. NMIBC = Non-muscle invasive bladder cancer; MIBC = Muscle-invasive bladder cancer. *p < 0.05; ****p < 0.0001

In our samples, we found that 43% (46/107) of the samples were diagnosed as low grade tumors and 57% (61/107) of the samples were diagnosed as high grade tumors. GANAB expression was found in 56 samples, including 8 low grade UCs and 48 high grade UCs. Only 17.4% (8/46) showed positive staining in low grade cases. Of the 61 high grade UCs, GANAB expression was found in up to 78.9% (48/61) cases. In general, the expression levels of GANAB were significantly increased in high grade tumors (Fig. 2A, B; and Table 1). Our data was consistent with the analysis of the TCGA-BLCA dada (Supplementary Table S3).

### Elevated expression levels of GANAB were correlated with advanced clinical stages of UC

In our cohort, 63 of the samples were diagnosed as early stage (T1/Ta) and 36 were MIBC. Of the 63 NMIBC tumors, 39.7% (25/63) showed positive expression of GANAB. Of the 36 MIBC tumors, positive GANAB expression was observed in 86.2% (31/36) samples. The expression level of GANAB was much higher in the MIBC samples as compared with the NMIBC samples (Fig. 2C, D).

**Fig. 3:**
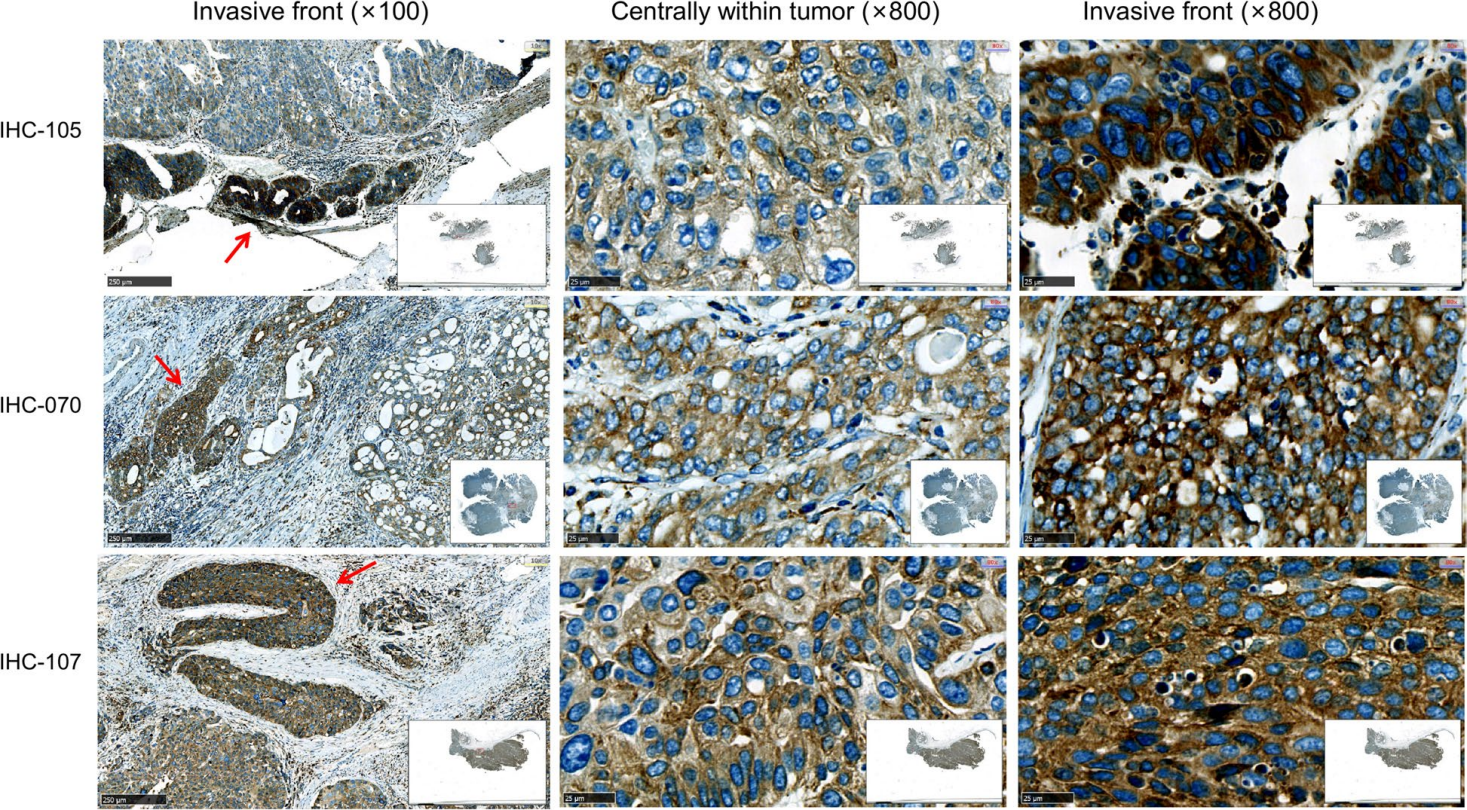
Tumor cells in UCs showed strong expression levels of GANAB in the invasive fronts. Left panel, the overview of invasive fronts at 100 × ; The expression of GANAB in the center of the tumor at 800 × (Middle); The elevated expression of GANAB in the invasive fronts at 800 × (Right panel). Scale bar: 250μm (Left panel). Scale bar: 25μm (Middle and Right panel). Arrows indicate the invasive fronts

Additionally, in 33.3% (12/36) MIBC cases, we found that the tumor cells at the invasive fronts of tumor margin exhibited much higher expression of GANAB than the tumor cells inside the tumor body, and more brown granules were observed at the invasive fronts. The results showed that GANAB might play a critical role in UC growth and invasion (Fig. 3; Table 1).

In the correlation analysis of GANAB with the clinical factors, we found that GANAB was significantly correlated with tumor cell mitosis (Ki67 as a marker), with p-value of 0.0319. No significant correlation was found for the vascular invasion (Supplementary Figure S1).

### GANAB promoted the proliferation in UC cells

**Fig. 4:**
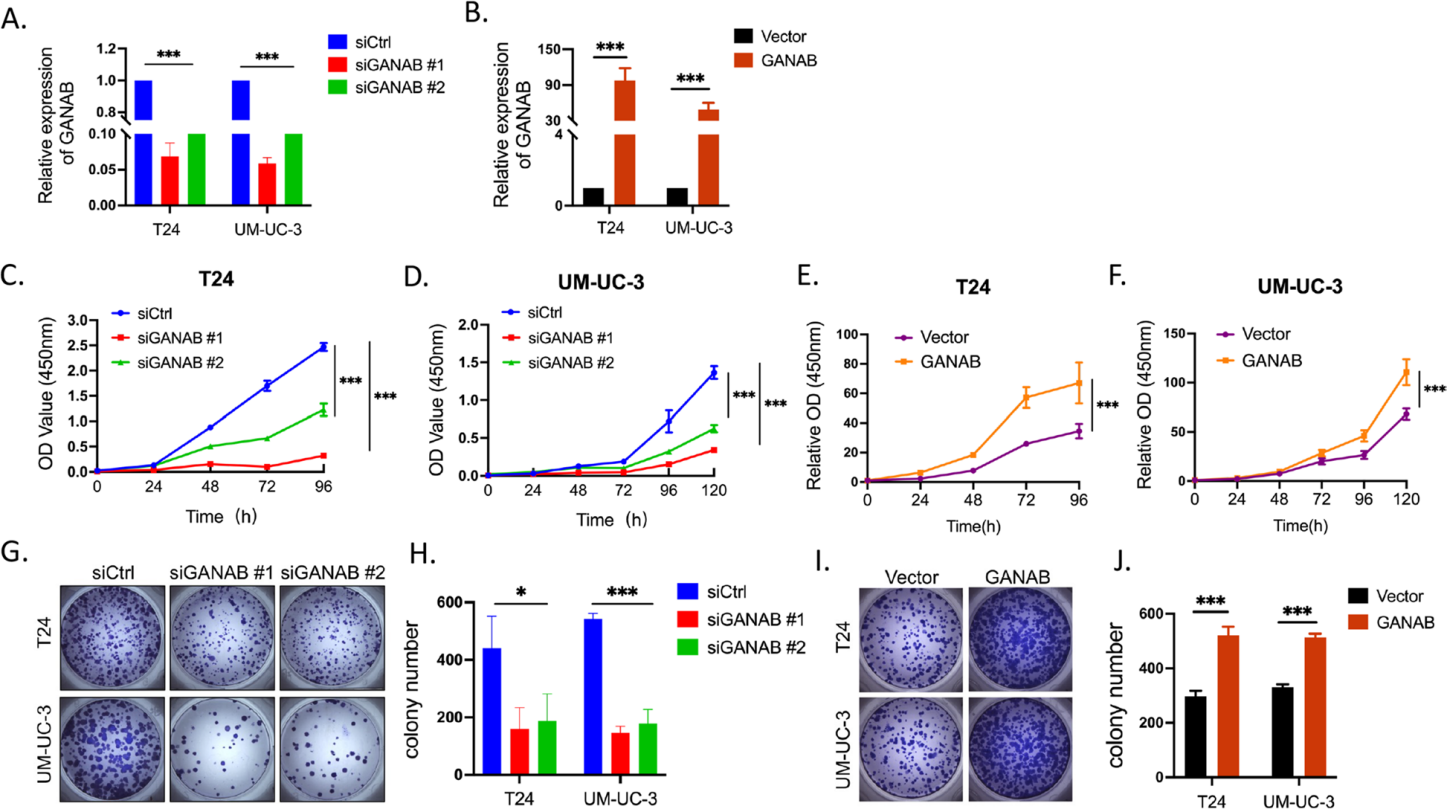
GANAB promoted the proliferation in UC cells. **A-B,** GANAB was markedly down-regulated or up-regulated after siGANABs or pcDNA3.1( +)-GANAB transfection. **C-D,** SiRNA knockdown of GANAB in T24 and UM-UC-3 cells significantly inhibited cell proliferation in CCK8 assay. **E–F,** Overexpression of GANAB in T24 and UM-UC-3 cells markedly increased cell proliferation in CCK8 assay. **G-H,** Colony-formation assays demonstrated that knockdown of GANAB dramatically inhibited the size and the number of colonies of T24 and UM-UC-3 cells. **I- J,** The overexpression of GANAB increased the size and the number of colonies of T24 and UM-UC-3 cells. *p < 0.05; ***p < 0.001

To explore the function of GANAB in UC cells, GANAB expression was silenced using RNAi and overexpressed by transfecting pcDNA 3.1( +)-GANAB. qRT-PCR showed that GANAB was remarkably down-regulated or up-regulated in UM-UC-3 and T24 cells after transfection (Fig. 4A, B). Cell proliferation was tested by CCK-8 assay. The results showed that the proliferation of UM-UC-3 and T24 cells in the silencing GANAB (siGANABs) group was significantly impaired compared with that in the control group (*p* < 0.001, Fig. 4C, D). Conversely, UC cells overexpressing GANAB exhibited a higher cell viability rate as compared with the controls cells (Fig. 4E, F). The colony-formation assays showed that siGANABs dramatically inhibited the sizes and the number of colonies in UC cells (Fig. 4G, H), while the colony-formation were markedly increased in the cell lines with GANAB overexpression (Fig. 4I, J). Taken together, these data indicated that GANAB played a vital role in the carcinogenesis of UC.

### GANAB promoted the migration and invasion in UC cells

**Fig. 5:**
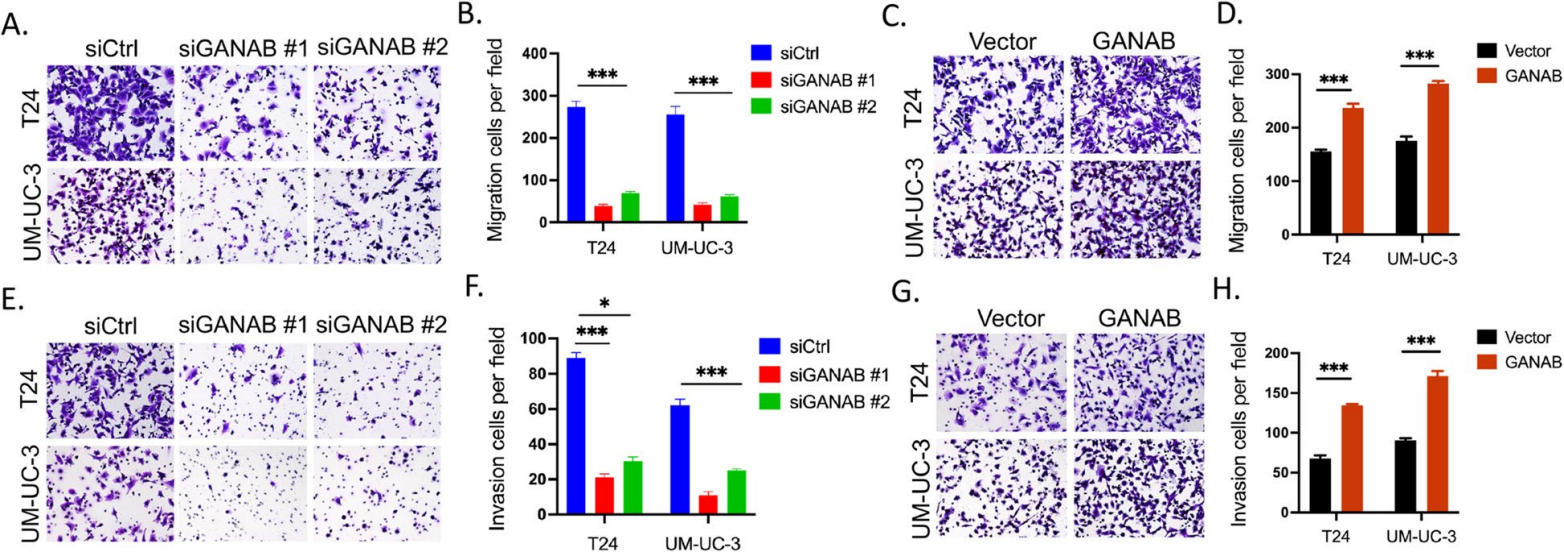
GANAB promoted the migration and invasion in UC cells. **A-B,** Silencing of GANAB reduced T24 and UM-UC-3 cells migration in Transwell assay. **C-D,** The overexpression of GANAB increased T24 and UM-UC-3 cells migration in Transwell assay. **E–F,** Silencing of GANAB reduced T24 and UM-UC-3 cells invasion in Transwell assay. **G-H,** The  overexpression of GANAB increased T24 and UM-UC-3 cells invasion in Transwell assay. *p < 0.05; ***p < 0.001

Furthermore, Transwell assay was performed to assess the effect of GANAB on the migration and invasion ability of UC cells. We found that the silencing of GANAB in UM-UC-3 and T24 cells caused a significant reduction in cell migration and invasion (Fig. 5A, B, E, F). Conversely, we found that overexpressing GANAB in UC cells promoted cell migration and invasion (Fig. 5C, D, G, H). These data indicated that GANAB played a vital role in the regulation of cell migration and invasion.

### Knock-down of GANAB induced cell cycle arrest

**Fig. 6:**
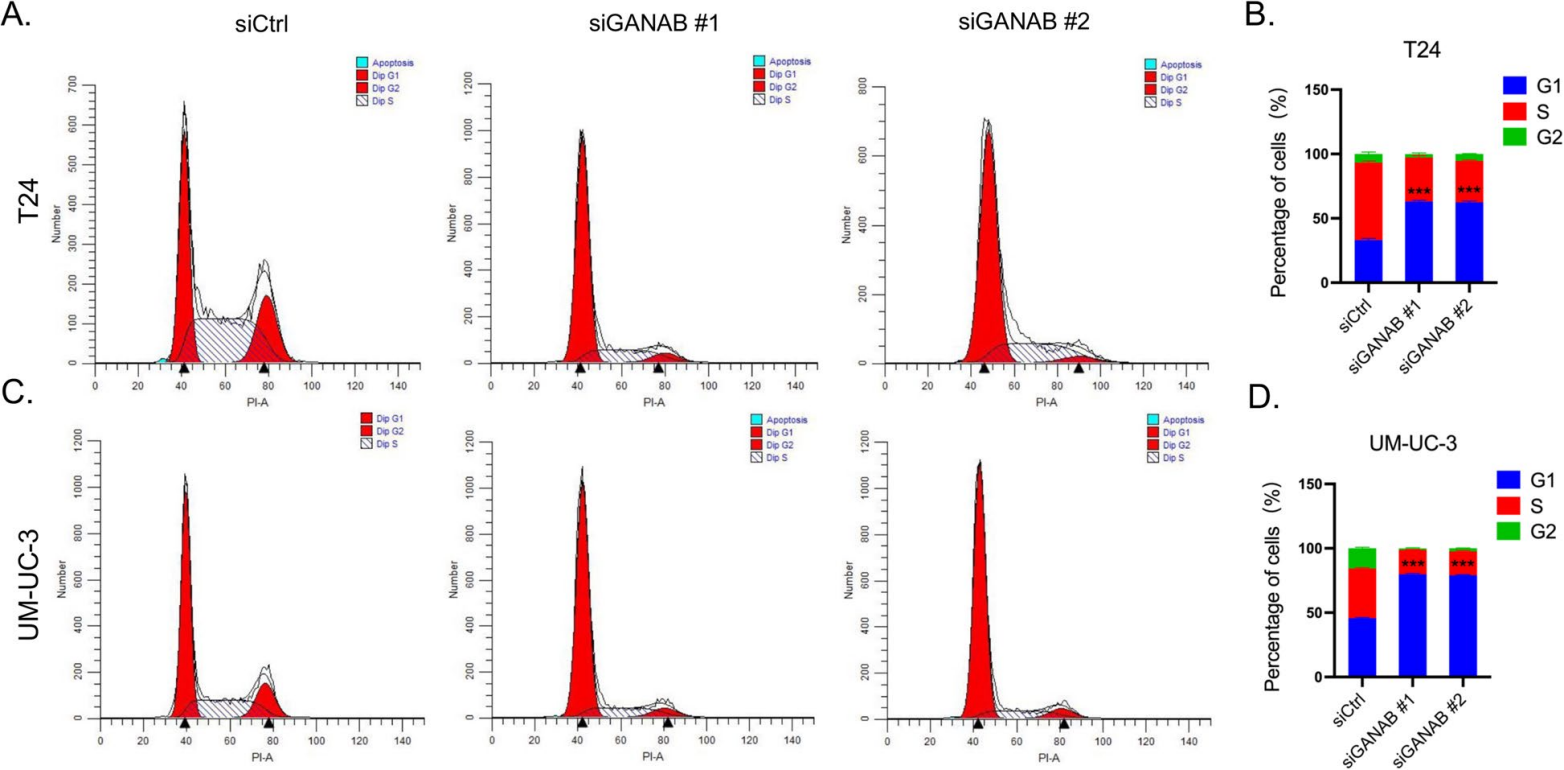
Knockdown of GANAB induced cell cycle arrest at the G1 phase in T24 and UM-UC-3 cells. **A-B**, T24 Cells of G1 phase were increased in the siGANABs group compared to the siCtrl group. **C-D**, UM-UC-3 Cells of G1 phase were increased in the siGANABs group compared to the siCtrl group. ***p < 0.001

Furthermore, cell cycle arrest was further detected by flow cytometry. Consistently, we observed that both of UM-UC-3 and T24 cells transfected with siGANABs showed a significant increase in the G1 phase as compared with that in the control cells (Fig. 6A, B, C, D). These results suggested that UC cells with GANAB knockdown were arrested at the G1 phase.

### Up-regulated GANAB expression levels were correlated with genomic amplifications and elevated hypoxia gene signature scores in clinical samples

**Fig. 7:**
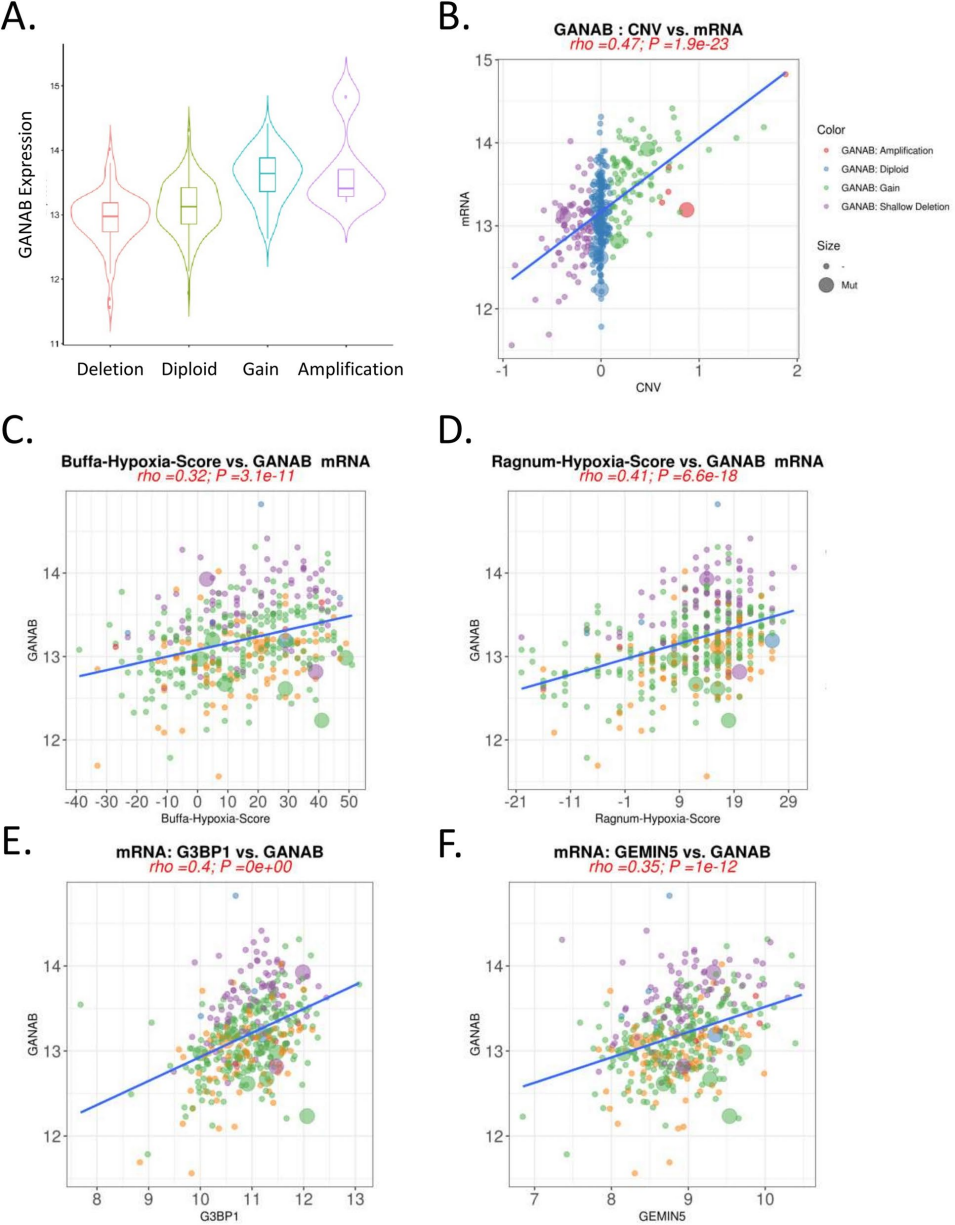
Bioinformatics analysis of the expression regulatory factors of GANAB. **A-B**, GANAB expression was up-regulated by the copy number amplifications of the gene loci of the UC genomes. **C-D**, The expression of the GANAB was positively correlated with the gene signature-derived Hypoxia scores in TCGA-BLCA data (CBioportal). **E–F**, The expression levels of the GANAB was positively correlated with Stress Granule (SG) factors (G3BP1 and GEMIN5). The color scheme used in **C-F** was the same as **B**. ‘-’, not profiled, or no mutation detected in the genomic data

To investigate the potential roles of the GANAB in cancer, we conducted correlation analyses of the gene expression and cancer attributes, including tumor grade, stage, recurrent somatic mutations, copy number alterations,and multiple gene signature scores. We found that GANAB expression levels were strongly correlated with its copy number amplifications in genomes in the TCGA/BLCA cohort. There was a clear trend that the up-regulated levels of GANAB were increased in copy number gains and amplifications, as compared to the shallow deletions and diploid (Fig. 7A, B). The copy number values computed by using the GISTIC software also showed a significant correlation with gene expression values (RSEM), with Spearman coefficient of 0.47 (*P* = 3.85 × 10^–28^). The results suggested that GANAB gene expression might be regulated by the copy number alternations in UCs (Fig. 7B).

Furthermore, we conducted an analysis on the correlation between the expression levels of GANAB and Hypoxia scores. We found that GANAB expression levels were significantly associated with Buffa hypoxia scores and Ragnum hypoxia scores in TCGA/BLCA samples. Moreover, we found that the expression levels of GANAB were closely correlated with G3BP1 (Spearman coefficient of 0.4, *P* = 1.57 × 10^–16^), which is an important hallmark of stress granule (SG) formation in cells. Similar results were found for GANAB *vs.* GEMIN5, both of which are located within G3BP1 genomic loci and also driven by copy number alterations in tumor (Fig. 7C-F; Supplementary Figure S2). The data indicated that the GANAB expression levels might be associated with hypoxia-mediated ER stress signaling (ERS) in clinical samples.

### Knockdown of GANAB induced ER stress and dys-regulation of cell cycle genes in *vitro*

**Fig. 8:**
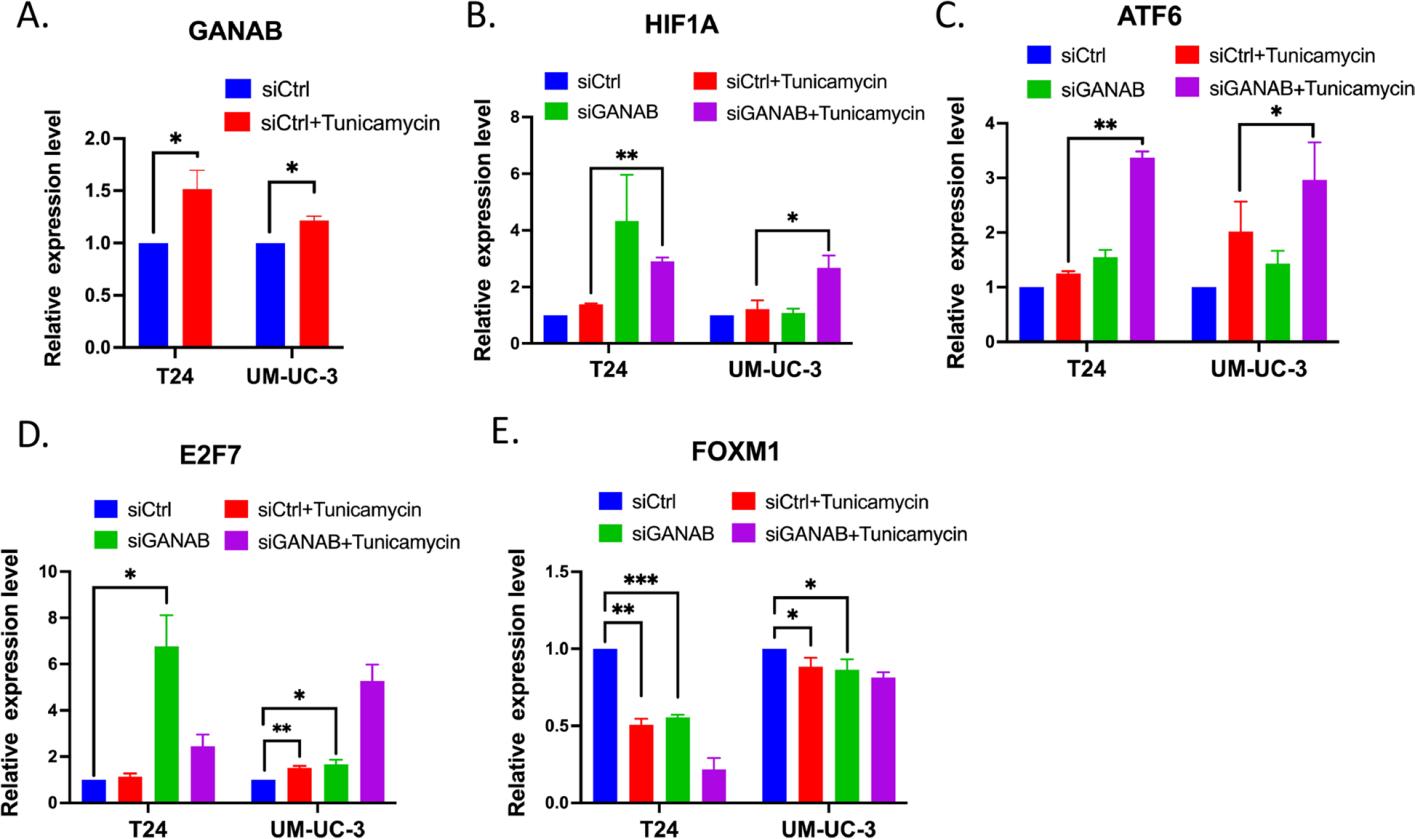
Knockdown of GANAB induced ER stress and dys-regulation of cell cycle genes in vitro. **A**, The expression of GANAB was up-regulated in T24 and UM-UC-3 cells with tunicamycin (Tm) stimulation. **B**, The expression of HIF1A in both siGANAB and Tm treatment was higher than that in cells with Tm treatment alone. **C**, The expression of ATF6 in both siGANAB and Tm treatment was higher than that in cells with Tm treatment alone. **D**, The expression of E2F7 was up-regulated in cells with siGANAB or Tm treatment. **E**, The expression of FOXM1 was down-regulated in siGANAB or Tm treatment. *p < 0.05, **p < 0.01, ***p < 0.001

According to the antibody manufacturer’s instructions, GANAB protein was located in ER sub-cellular structures. To determine whether GANAB was involved in the regulation of ERS signaling, we conducted experiments to quantitate the transcriptional activation of ATF6 by using siRNA-mediated GANAB knockdown in bladder cancer cell lines with/without tunicamycin (Tm) stimulation. According to previous studies, ATF6 activation is one of the critical signals of ERS [14] and G3BP1 is a marker of SG formation [15]. Interestingly, we found that Tm treatment induced the up-regulation of GANAB expression (Fig. 8A). Moreover, the obligation of GANAB resulted in similar effects of Tm stimulation of ERS in the T24 and UM-UC-3 cell line. We observed the consistent up-regulation of HIF1A, ATF6 in both si-GANAB and Tm treatment, suggesting the activation of ERS signaling in GANAB knockdown. Notably, si-GANAB synergistically enhanced the effects of Tm in our in vitro models (Fig. 8B, C).

In addition, to investigate the potential mechanisms of GANAB participating in the regulation of cell cycle, we included two cell cycle regulators, namely, E2F7 and FOXM1, in the qRT-PCR assays. Previous evidence has established that E2F7 transcriptional factor plays an essential role in the regulation of cell cycle progression; FOXM1 encoded protein is activated in M phase and has been validated as an essential transcriptional factor playing roles in bladder cancer progression [16]. The data showed that similar to Tm treatment, knockdown of GANAB enhanced the expression of E2F7, but inhibited the expression of FOXM1 (Fig. 8D; E). Further studies are warranted for understanding the regulatory axes of GANAB to FOXM1/ E2F7.

###  Functional network analysis of GANAB

**Fig. 9:**
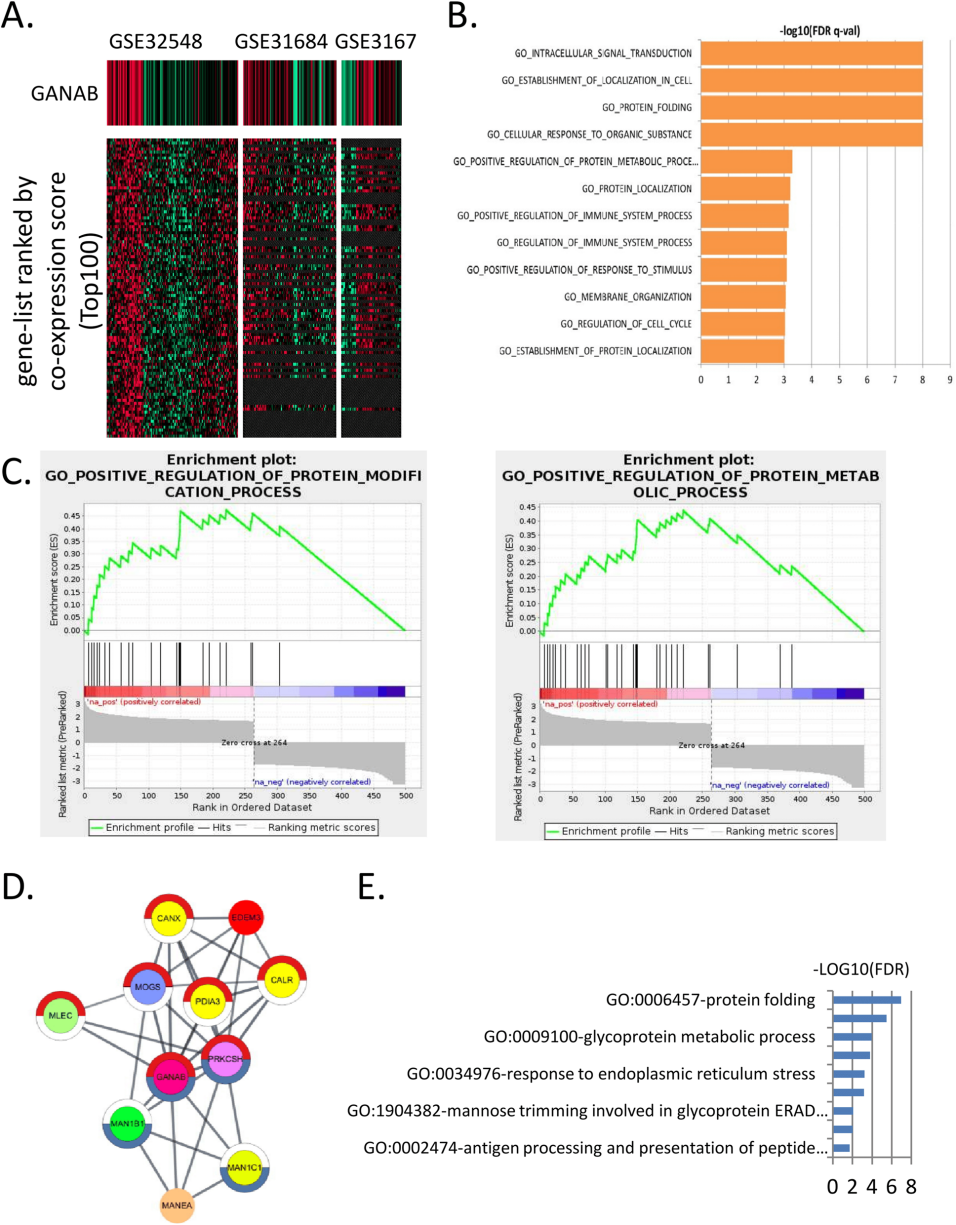
Enrichment analysis of GANAB correlated genes in bladder cancer microarray datasets. **A**, Co-expression heatmap of GANAB correlated genes in SEEK analysis. **B**, SEEK data analysis of tumor microarray data showed significant enrichment of ER stress and glycoprotein metabolic process correlated with GANAB gene expression. **C**, The enrichment plots for the specific pathway/networks. **D**, Network visualization of GANAB-related genes (STRING network) in Cytoscape. The Protein folding (GO:0006457) genes were bordered in red in the circle chart, and the N-glycan processing (GO: 0006491) genes were bordered in blue. **E,** The enrichment analysis of the GANAB network showed significant enrichments in the protein folding, glycoprotein metabolic process and ER stress signaling pathways

Finally, to investigate the roles of the GANAB co-expressed genes in UC, by using the SEEK bioinformatics tools, we conducted a co-expression analysis and found that the top-ranked co-expressed genes of GANAB were significantly enriched in the up-regulated signals in the pathways of ER stress, glycoprotein metabolic process and post-translational protein modification (Fig. 9A, B). GSEA Enrichment analysis indicated that the co-expressed gene networks were significantly associated with the biological processes of protein folding, glycol-protein metabolic process (Fig. 9C). The results were consistently with our findings in the SEEK analysis. To investigate the potential biological functions of GANAB-related genes, we conducted an enrichment analysis based on the PPI database in StringDB. We found that GANAB was strongly interacted with manose metabolism enzymes, ER stress and cell-cycle related protein CALR (Fig. 9D, E). The PPI interaction networks showed highly consistent of the findings in our correlation analyses.

## Discussion

A growing body of literatures dedicated to discovering the prognostic biomarkers for UC. Although many prognostic biomarkers have been reported in previous researches, it is difficult to predict the prognosis of UC due to the substantial tumor heterogeneity. It has been acknowledged that UC can be grouped into several different molecular subtypes, which seems to be correlated to the patients’ prognosis [17]. However, molecular stratification of UCs is far from being put into practice because of the high costs and the requirements of high-level expertise. It is urgently needed to excavate out a feasible and valuable prognostic biomarker for UC. In the present study, we reported GANAB as a novel biomarker with a significant prognostic value for UC. The expression of GANAB, an indicator of poorer prognosis, was further validated to be increased with tumor grades, tumor stages, and luminal subtypes of UC, indicating that GANAB might be attributed to the pathogenesis of UC. Moreover, in vitro experiments by knocking down of GANAB inhibited cell proliferation and migration. Knockdown of GANAB resulted in cell cycle arrest at G1 phase.

Our results were consistent with the previous findings in other cancers. Qin et al. [18] revealed that the expression levels of GANAB mRNA were higher in gastric cancer (GC) than the normal gastric tissues. In addition, it was found that RNA interference-mediated silencing of GANAB suppressed cell proliferation and promoted cell apoptosis in GC cell lines. However, we didn’t find obvious apoptosis after knocking down of GANAB (data not shown). In addition, it was found that higher expression levels of GANAB were intimately associated with poor prognosis in melanoma [19]. In addition, we observed that the expression levels of GANAB elevated in UC as compared with normal controls from TCGA/BLCA dataset and NCBI-GEO (GSE3167, GSE38264) datasets (Supplementary Figure S3).

 Previous studies reported that the heterogeneity and functional diversity within cell populations occur owing to aberrant protein glycosylation, which might be protein-specific, site-specific and cell-specific [20]. A recent study identified several glycosylation sites in different breast cancer subtypes, which might be helpful for revealing the relationship between glycosylation and heterogeneity in breast cancer [21]. Dr. Anand Mehta. et al. demonstrated a correlation of specific sugar structures to specific hepatocellular carcinoma subtypes [22]. Further studies on the relationship of GANAB-mediated aberrant glycosylation and UC molecular subtypes might be able to explain the heterogeneity of UC.

In addition to playing roles in tumor cell migration and invasion [23, 24], the aberrant expression of glycosylation system might be correlated to immune response in cancer. For example, PD-L1 glycosylation was reported to play an oncogenic role in cancer immune evasion. N-glycosylated PD-L1 was found in melanoma, breast cancer, lung cancer, and colon cancers [25]. Glycosylated PD-L1 is largely unknown in UC and warrants future investigations.

The underlying mechanisms of GANAB in cancer are unclear. Previous studies revealed that GANAB gene encodes the GIIα, which is a neutral glucosidase located in the ER involved in N-linked Glycosylation. The process of N-linked glycosylation plays a role in protein folding, maturation and trafficking of membrane and secreted proteins in the ER [18, 26, 27]. Disruption of GANAB led to the accumulation of mis-folded glycosylation and the induction of the unfolded protein response [26]. In the polycystic liver disease condition, the hepatocystin fails to assemble with GANAB during carbohydrate processing, leading to altered cellular proliferation and differentiation [28]. Recent studies showed that GANAB was involved in ER stress [29], and regulation of protein folding was the most important process for GANAB [30]. Taken together, the essential biological functions of GANAB might lie in the process of protein-folding in ER stress. Although our in vitro experiments showed that knockdown of GANAB resulted in cell cycle arrest and led to inhibition of cell proliferation and metastasis, the underlying mechanism connecting GANAB and protein-folding in ER is unknown. A previous study indicated that tunicamycin(Tm) could specifically inhibit DPAGT1 and induce substantial ERS [31]. DPAGT1 is the enzyme that catalyzes the first step of protein N-glycosylation, involving in the same metabolic pathway as GANAB. Our data suggested that GANAB mediated the activation of ER stress signaling including the transcriptional activation of ATF6 and HIF1A. Moreover, GANAB might participate in the regulation of ER stress-related cell cycle by modulating transcriptional factors such as E2F7 and FOXM1. Interestingly, we found that siGANAB synergistically enhanced the effects of tunicamycin in our in vitro models, suggesting that GANAB might serve as a promising therapeutics target for bladder cancer that warrant further studies.

Based on our results and literature rationales, the roles of GANAB in the proliferation and migration of UC tumor cells might be mediated by the aggregation of SGs during ER stress (Supplementary Figure S4). In tumor cells, frequent TP53 mutations, G3BP1 amplification and GEMIN5 amplification, might co-ordinate with the amplification of GANAB in cancerous genomes. During ER stress, protein translation was arrested and subjected to quality control (QC) by PTMs. As a result, the RNA-binding proteins aggregate and assemble into SGs. Such processes might promote tumor cell proliferation and migration in a yet known mechanism. The mechanisms of interaction between GANAB and ER stress in UC required further experimental investigations.

In addition to GANAB, there are numerous N-glycosylation-associated markers that were reported being strongly associated with tumor progression such as DPAGT1 [32]. Morever, overexpression of GnT-V glycosyltransferase enhances CEACAM6 N-glycosylation to predict recurrence of oral squamous cell carcinoma [33]. A study showed that up-regulation of glycosylation-related enzyme, alpha (1,6) fucosyltransferase (FUT8) in castration-resistant prostate cancer (CRPC) resulted in an increase of EGFR and leading to increased cell survival [34]. Therefore, future studies on GANAB and other N-glycosylation-associated proteins in UCs are needed to elucidate the unknown mechanisms of protein-glycosylation in the pathogenesis of UC.

## Conclusion

In summary, we found that the elevated expression levels of GANAB were correlated with poorer overall survival of UCs. Our studies recognized that GANAB, an aberrantly protein-glycosylation protein might serve as a promising molecular biomarker for risk stratification of UC. In vitro experiments showed that GANAB knockdown resulted in cell cycle arrest and led to inhibition of cell proliferation and metastasis. Our studies highlighted the important roles of GANAB mediated glycosylation process in the ER stress and SG formation in UCs that warrants further investigations.

## Supplementary Information

** Additional file 1: Supplementary Figure S1. **Comparisons of GANAB gene expression (H-scores)stratified by clinical variables. A, The expression of GANAB in patients with high and low expression of KI-67. B, The expression of GANAB in patients withlarge (≥3mm) and small (<3mm) tumor size. C, The expression of GANAB in patients with/without vascular invasion. **Supplementary Figure S2. **Pan-cancer analysis of the expression levels of GANAB in correlation with G3BP1 and GEMIN5 expression. A, GANAB gene expression was positively correlated with G3BP1 expression. B, GANAB gene expression was positively correlated with GEMIN5 expression. **Supplementary Figure S3. **Expression levels of GANAB in tumors compared with the normal tissues. GSE3167,GSE38264 and TCGA-BLCA datasets were used for analysis. A, The expression of GANAB was upregulated in UC tissues as compared with the non-tumor tissues in GSE3167 dataset. B, The expression of GANAB was upregulated in UC tissues as compared with the non-tumor tissues in GSE38264 dataset. C, The expression of GANAB was upregulated in UC tissues as compared with the non-tumor tissues in TCGA-BLCA dataset (TCGA/GEPIA). **Supplementary Figure S4.** The schematics shows the potential roles of GANAB in the proliferation and migration of UC tumor cells mediated by ER stress. Briefly, tumor cells frequently harbor G3BP1 amplification and GEMIN5 amplifications, coordinated with the amplification of GANAB in cancerous genomes. During ER stress, stress-related transcriptional factors HIF1A, ATF6 are increased. The roles of GANAB in the proliferation and migration of UC tumor cells might be mediated by participating in the regulation of ER stress signaling such as ATF6 pathway and stress-related cell cycle by modulating the activities of transcriptional factors.

**Additional file 2: Supplementary Table S1.** Survival analysis of the glycoproteinscorrelated with prognosis of the UCs in TCGA data.

**Additional file 3: Supplementary Table S2.** Full results of the Univariate analysis ofglycogenes correlated with UC prognosis in TCGA/BLCA cohort.

**Additional file 4: Supplementary Table S3.** Correlation analysis of clincal factors andGANAB gene expression in TCGA-BLCA dataset.

## Data Availability

All data generated or analyzed during this study are included in this article and referenced articles are listed in the References section. The TCGA Gene expression data and corresponding clinical information were obtained from the Cancer Genome Atlas (TCGA, https://tcga-data.nci.nih.gov/tcga/). Array- based gene expression data were obtained from Gene Expression Omnibus (GEO), including GSE32548 (https://www.ncbi.nlm.nih.gov/geo/query/acc.cgi?acc=GSE32548, GSE31684 (https://www.ncbi.nlm.nih.gov/geo/query/acc.cgi?acc=GSE31684), and GSE3167 (https://www.ncbi.nlm.nih.gov/geo/query/acc.cgi?acc=GSE3167). The SEEK bioinformatics tools was used for co-expression analysis accessed through https://seek.princeton.edu/seek/.

## Abbreviations

UC: Urothelial carcinoma
NMIBC: Non-muscle invasive bladder cancer
MIBC: Muscle-invasive bladder cancer
PTMs: Post-translational modifications
ER: Endoplasmic reticulum
SG: Stress granule
GluII: Glucosidase II
GIIα: α-Subunit of glucosidase II
IHC: Immunohistochemistry
WSI: Whole slide image
FFPE: Formalin-fixed paraffin-embedded
ROIs: Regions of Interest
GEO: Gene expression omnibus
TCGA: The Cancer Genome Atlas
qRT-PCR: Quantitative real-time PCR
GAPDH: Glyceraldehyde 3-phosphate dehydrogenase
siRNA: Small interfering RNA
siCtrl: Silencing control
