# Supplementary material for: Glycoprotein α-Subunit of Glucosidase II (GIIα) is a novel prognostic biomarker correlated with unfavorable outcome of urothelial carcinoma
Source: BMC Cancer. 2022 Jul 25;22:817. doi: 10.1186/s12885-022-09884-8 (PMC9316353; doi:10.1186/s12885-022-09884-8)
Supplement: Supplementary file 1 — Additional file 1: Supplementary Figure S1. Comparisons of GANAB gene expression (H-scores)stratified by clinical variables. A, The expression of GANAB in patients with high and low expression of KI-67. B, The expression of GANAB in patients withlarge (≥3mm) and small (<3mm) tumor size. C, The expression of GANAB in patients with/without vascular invasion. Supplementary Figure S2. Pan-cancer analysis of the expression levels of GANAB in correlation with G3BP1 and GEMIN5 expression. A, GANAB gene expression was positively correlated with G3BP1 expression. B, GANAB gene expression was positively correlated with GEMIN5 expression. Supplementary Figure S3. Expression levels of GANAB in tumors compared with the normal tissues. GSE3167,GSE38264 and TCGA-BLCA datasets were used for analysis. A, The expression of GANAB was upregulated in UC tissues as compared with the non-tumor tissues in GSE3167 dataset. B, The expression of GANAB was upregulated in UC tissues as compared with the non-tumor tissues in GSE38264 dataset. C, The expression of GANAB was upregulated in UC tissues as compared with the non-tumor tissues in TCGA-BLCA dataset (TCGA/GEPIA). Supplementary Figure S4. The schematics shows the potential roles of GANAB in the proliferation and migration of UC tumor cells mediated by ER stress. Briefly, tumor cells frequently harbor G3BP1 amplification and GEMIN5 amplifications, coordinated with the amplification of GANAB in cancerous genomes. During ER stress, stress-related transcriptional factors HIF1A, ATF6 are increased. The roles of GANAB in the proliferation and migration of UC tumor cells might be mediated by participating in the regulation of ER stress signaling such as ATF6 pathway and stress-related cell cycle by modulating the activities of transcriptional factors. [file 12885_2022_9884_MOESM1_ESM.pdf]

Supplementary Figures

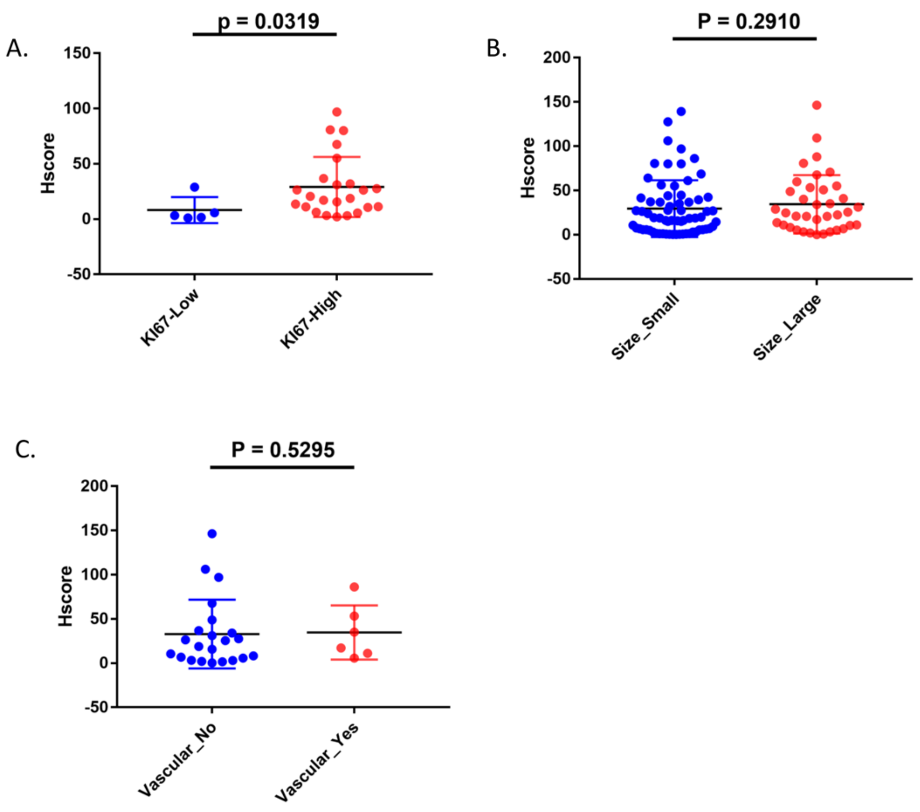

Supplementary Figure S1. Comparisons of GANAB gene expression (H-scores) stratified by clinical variables. A. The expression of GANAB in patients with high and low expression of ki-67. B. The expression of GANAB in patients with large (≥3mm) and small (<3mm) tumor size. C. The expression of GANAB in patients with/without vascular invasion.

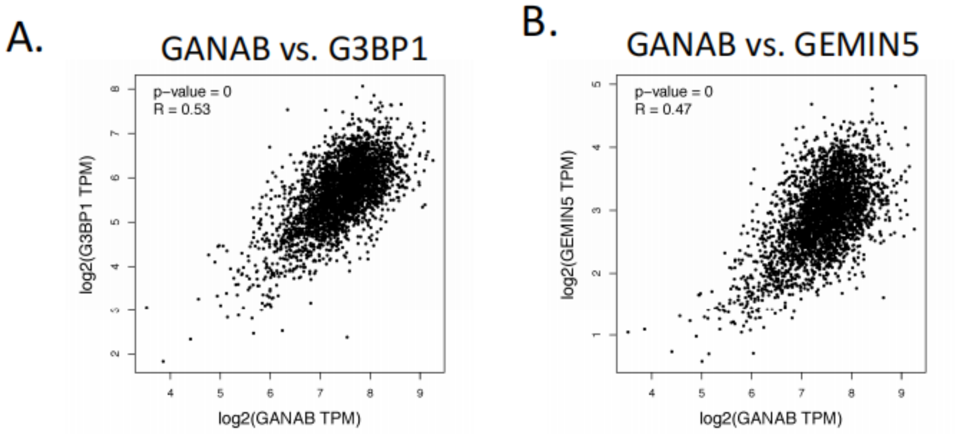

Supplementary Figure S2. Pan-cancer analysis of the expression levels of GANAB were strongly correlated with G3BP1 and GEMIN5, a hallmark of the SG formation. A. GANAB expression level was positively correlated with G3BP1 expression level. B. GANAB expression level was positively correlated with GEMIN5 expression level.

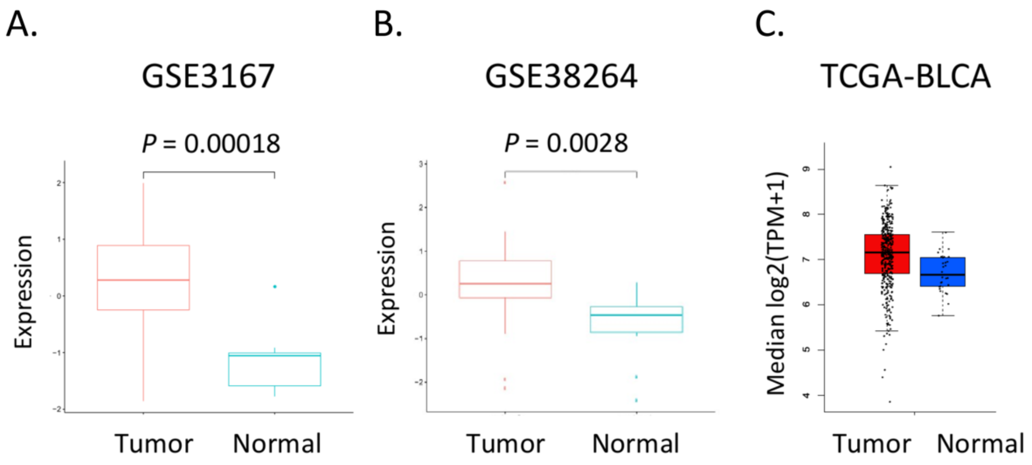

Supplementary Figure S3. Elevated expression levels of GANAB in tumors as compared with normal controls. GSE3167, GSE38264 and TCGA-BLCA datasets were used for analysis. A. The expression of GANAB is upregulated in UC tissues as compared with the non-tumor tissues in GSE3167 dataset. B. The expression of GANAB is upregulated in UC tissues as compared with the non-tumor tissues in GSE38264 dataset. C. The expression of GANAB is upregulated in UC tissues as compared with the non-tumor tissues in TCGA-BLCA dataset.

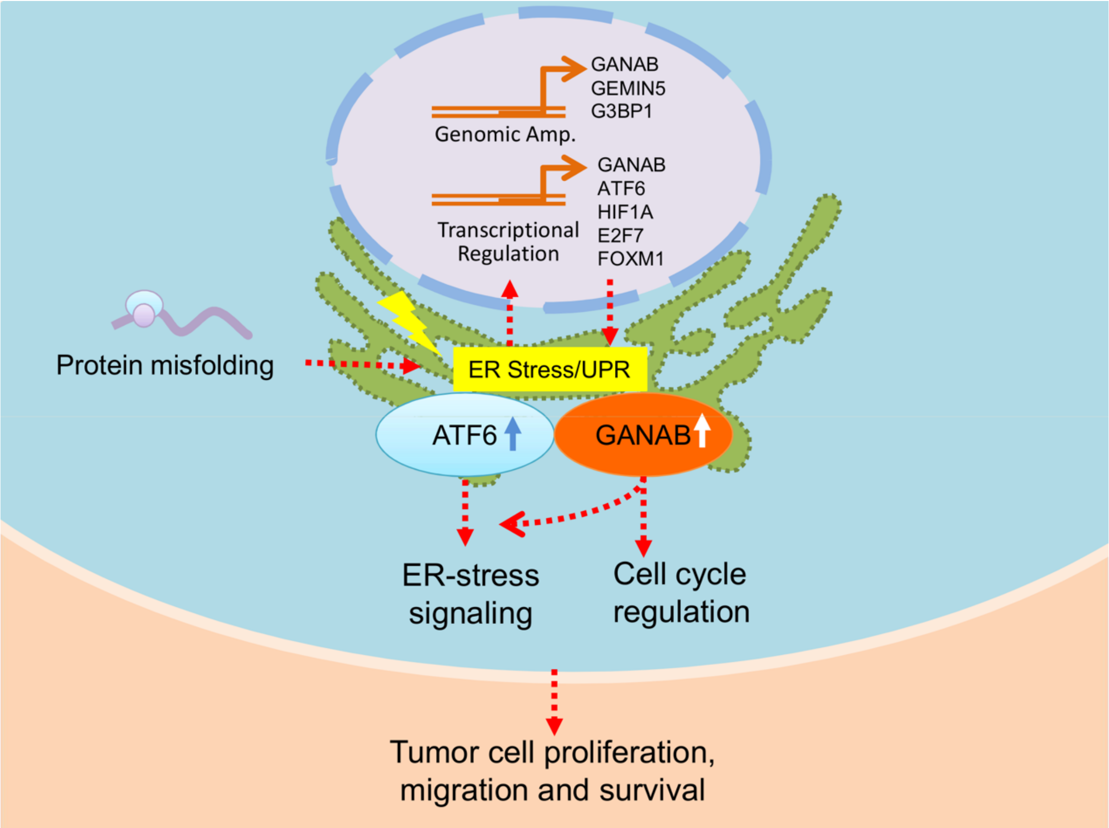

Supplementary Figure S4. The schematics shows the potential roles of GANAB in the proliferation and migration of UC tumor cells mediated by ER stress. Briefly, tumor cells frequently harbor G3BP1 amplification and GEMIN5 amplifications, coordinated with the amplification of GANAB in cancerous genomes. During ER stress, stress-related transcriptional factor HIF1A, ATF6 are increased. The roles of GANAB in the proliferation and migration of UC tumor cells might be mediated by participating in the regulation of ER stress signaling such as ATF6 pathway and related cell cycle by modulating the activities of transcriptional factors.
